# *Geum japonicum* Thunb. exhibits anti-platelet activity via the regulation of cyclic guanosine monophosphate

**DOI:** 10.3389/fphar.2025.1538417

**Published:** 2025-06-26

**Authors:** Yuan Yee Lee, Abdul Wahab Akram, Young-Hee Kim, Muhammad Irfan, Sung Dae Kim, Evelyn Saba, Tae Wan Kim, Bong-Sik Yun, Man Hee Rhee

**Affiliations:** ^1^ Department of Veterinary Medicine, Kyungpook National University, Daegu, Republic of Korea; ^2^ Department of Animal and Avian Sciences, University of Maryland, College Park, MD, United States; ^3^ Natural Products Chemistry R&D Department, Hongcheon Institute of Medicinal Herb, Hongcheon-gun, Gangwon, Republic of Korea; ^4^ Department of Oral Biology, University of Illinois at Chicago, Chicago, IL, United States; ^5^ Department of Veterinary Biomedical Sciences, Faculty of Veterinary and Animal Sciences, Pir Mehr Ali Shah Arid Agriculture University, Rawalpindi, Pakistan; ^6^ Division of Biotechnology and Advanced Institute of Environment and Bioscience, College of Environmental and Bioresource Sciences, Jeonbuk National University, Iksan, Republic of Korea; ^7^ Institute for Veterinary Biomedical Science, College of Veterinary Medicine, Kyungpook National University, Daegu, Republic of Korea

**Keywords:** platelet aggregation, cardiovascular disease, *Geum japonicum*, cyclic guanosine monophosphate, anti-platelet

## Abstract

**Introduction:**

Traditionally, *Geum japonicum* Thunb. (GJ) extract has been used to treat headaches and dizziness. We hypothesize that GJ exhibits anti-platelet activity that may prevent ischemic events to alleviate these symptoms. In this study, we investigated the anti-platelet activity of GJ as a potential mechanism for enhancing blood flow and preventing vessel occlusion.

**Methods:**

Platelets were stimulated with collagen, adenosine diphosphate (ADP) or thrombin. Platelet aggregation was carried out using a platelet aggregometer with washed platelets from Sprague-Dawley rats. We observed the mobilization of calcium ions using Fura-2AM and adenosine triphosphate (ATP) release via a luminometer. The activation of integrin αIIbβ3 and population of platelet-neutrophil aggregates (PNAs) were investigated using flow cytometry. Platelet shape change was observed using scanning electron microscopy and transmission electron microscopy.

**Results:**

GJ extract inhibited collagen, ADP and thrombin-induced platelet aggregation. It effectively prevented the mobilization of calcium ions, ATP secretion, and serotonin release while thromboxane B2 levels did not change. Moreover, GJ inhibited the inside-out and outside-in signaling of integrin αIIbβ3. Notably, GJ treatment led to elevated expression of cyclic guanine monophosphate (GMP) (but not cyclic adenosine monophosphate). The protein expressions in the PI3K/Akt pathway were inhibited and platelet shape change was prevented. Finally, GJ treatment resulted in a decreased population of PNAs *in vivo*.

**Discussion:**

GJ exhibits potent anti-platelet activity acting by upregulating cGMP. It holds promise as a potential candidate for supplementation in patients with cardiovascular disease and thrombosis.

## 1 Introduction

Platelets, the smallest blood cells in the human body, originate from megakaryocytes in the bone marrow. When a vessel is damaged, it exposes the subendothelial matrix, recruiting platelets to form a hemostatic plug that prevents further vessel wall damage (Jurk and Kehrel, 2005). Collagen release from the subendothelial layer during vessel injury activates platelets via the glycoprotein VI (GPVI) receptor (Sangkuhl et al., 2011). This activation leads to platelet shape change and the secretion of granules, including adenosine diphosphate (ADP) and thromboxane A2 (TXA2) (Nieswandt et al., 2003). As reported, ADP also acts as an agonist of the P2Y1 and P2Y12 receptors, further activating platelets (Hardy et al., 2005). Additionally, integrin αIIbβ3 undergoes conformational changes, allowing platelets to aggregate in the presence of fibrinogen (Ye et al., 2011). Thrombin generated locally, activates platelets via the protease-activated receptor, contributes to the formation of a stable hemostatic plug or thrombus (Brass, 2003).

Inflammation within blood vessels triggers the secretion of inflammatory cytokines and adhesion molecules, including monocyte-chemoattractant factor 1 (MCP-1) (Goede et al., 1999; Ohlsson et al., 2009). Tumor necrosis factor-α stimulates MCP-1, attracting monocytes to the vessel wall (Murao et al., 2000). Additionally, intracellular adhesion molecule-1 (ICAM-1) and vascular cell adhesion molecule-1 (VCAM-1) also play a role in monocyte recruitment to the vascular endothelium (Walpola et al., 1995). Oxidative damage caused by reactive oxygen species to the endothelial lining converts low-density lipoprotein (LDL) into oxidized LDL (oxLDL) (Matsuura et al., 2008; Stocker and Keaney, 2004). Monocytes that are attracted by MCP-1 migrate into the vascular endothelium, differentiate into macrophages, internalize oxLDL, forming foam cells (Bobryshev, 2006). These foam cells contribute to the formation of a necrotic core (Stocker and Keaney, 2004), while their secretion of matrix metalloproteinases degrades the extracellular matrix (Newby et al., 2009) ultimately leading to the development of a vulnerable plaque.

Vessel occlusion due to plaque formation, can cause ischemic stroke due to restricted blood flow to the brain (Lee et al., 2018), and thrombosis may contribute to headaches due to temporary disruptions of blood flow. To prevent thrombus formation and vessel occlusion, one crucial intervention is inhibiting platelet activation. Platelets expresses various receptors on the membrane and can form aggregates not only with other platelets (Jackson, 2007), but also with leukocytes (Totani and Evangelista, 2010) and monocytes (Freedman and Loscalzo, 2002). Therefore, preventing platelet aggregation becomes a vital intervention to prevent stroke.

*Geum japonicum* Thunb. (GJ), also known as the Asian herb bennet, is locally referred to as 뱀무 (*Baem-mu*) in Korea. GJ was used as a diuretic, an astringent, and a remedy for headache and dizziness (Liu et al., 2009). Previous research has highlighted its anti-HIV activities (Xu et al., 1996) and anti-depression properties (Lim et al., 2019). Purified tannins from GJ exhibit hypotensive effects (Xie et al., 2007) and possess anti-angiogenic properties that may be beneficial in muscle ischemia (Cheung et al., 2007). Additionally, GJ was also reported to inhibit fatty acid synthases (Liu et al., 2009). Moreover, GJ has been investigated for its ability to repair infarcted myocardium by promoting early revascularization and myocardial regeneration (Li et al., 2006). It was also reported to inhibits tumor metastasis (Heo et al., 2008) while restoring occluded coronary vessels and reconstituting the coronary vasculature (Chen et al., 2014). Given that the vascular endothelium plays a crucial role in thrombosis and vessel occlusion due to its various adhesion molecules and platelet aggregation agonists, we are investigating GJ’s potential to inhibit platelet aggregation as a part of its anti-angiogenic effects.

Recent studies have shown that platelets form aggregates with neutrophils, resulting in platelet-neutrophil aggregates (PNAs). Neutrophils accumulate at injury sites during atherosclerotic plaque rupture, where activated platelets interact with neutrophils via adhesion molecules (Pircher et al., 2019). The circulating PNAs have been implicated in conditions such as deep venous thrombosis (Zhou et al., 2019), ischemic stroke (Denorme et al., 2021) and type 1 diabetes (Popp et al., 2022). Platelets have also been observed to aggregate with monocytes and lymphocytes (Finsterbusch et al., 2018). Our investigation focused on PNAs as they contribute to the formation of neutrophil extracellular traps (NETs), which exacerbate tissue damage (Carestia et al., 2016). Thus, platelet activation and aggregation can lead to a cascade of undesirable pathologies, especially when paired with their ability to form aggregates with leukocytes like neutrophils.

Our study demonstrated that GJ effectively inhibits platelet aggregation induced by collagen, ADP, and thrombin. Further investigation into its mechanism of action revealed that GJ upregulates cyclic guanosine monophosphate (cGMP) and inhibits the phosphoinositide 3-kinase/protein kinase B (PI3K/Akt) and mitogen-activated protein kinase (MAPK) pathways, in addition to preventing the conformational change of integrin αIIbβ3. However, further studies are needed to elucidate its anti-platelet activity *in vivo*. Notably, our study shows that GJ’s anti-platelet activity, mediated by cGMP upregulation, may contribute to preventing thrombosis and vessel occlusion.

## 2 Materials and methods

### 2.1 Acquisition and preparation of GJ extract

The whole plant of GJ was obtained from the Natural Product Central Bank (Ochang-eup, Cheongju-si, Chungcheongbuk-do, Republic of Korea) registered with voucher number KPM032-039 (https://www.kobis.re.kr/npcb/uss/main.do). Dried whole plant of GJ was extracted using 50% ethanol for 2 h at 80°C at a ratio of 1:20 (dried plant: solvent) in a reflux setup to minimize evaporation. After extraction, the extract was filtered with Grade 1 Whatman^®^ filter paper (GE Healthcare, PA, United States) and concentrated using a rotary evaporator (Rotavapor^®^ R-100; BUCHI Labortechnik, Switzerland) to remove excess solvent. The extract was frozen and lyophilized to obtain the solvent-free powdered extract that was stored at −30°C until use. Powdered extract of GJ was weighed and dissolved in dimethylsulfoxide (DMSO) for subsequent experiments.

### 2.2 Ultra-performance liquid chromatography and quadrupole time-of-flight mass spectrometry (UPLC-QTOF-MS) analysis of GJ

UPLC–QTOF-MS analysis was performed using an Acquity H-Class Plus UPLC system (Waters Corporation, Milford, MA, United States) interfaced with an Acquity RDa QTOF-MS detector (Waters). Chromatographic separation was achieved using an Acquity UPLC HSS C18 reverse-phase column (2.1 × 100 mm, 1.8 µm particle size; Waters). The mobile phase consisted of solvent A (distilled water containing 0.1% formic acid) and solvent B (acetonitrile containing 0.1% formic acid). A linear gradient elution was performed, starting with 10% solvent B for 2 min, increasing to 100% over 25 min, and held at 100% for an additional 5 min. The flow rate was maintained at 0.2 mL/min, and the injection volume was 1 µL. Mass spectrometric detection was carried out in negative electrospray ionization (ESI) mode, with a desolvation temperature of 550°C, a capillary voltage of 0.8 kV, and a cone voltage of 30 V.

### 2.3 Chemicals and reagents

Collagen, ADP and thrombin were obtained from Chrono-Log (Havertown, PA, United States). The adenosine triphosphate (ATP) assay kit was from the Biomedical Research Service (Buffalo, NY, United States). Enzyme-linked immunoassay (ELISA) kits for thromboxane B2 (TXB2; item no. 501020), cyclic adenosine monophosphate (cAMP; item no. 501040), and cGMP (item no. 581021) were obtained from Cayman Chemicals (Ann Arbor, MI, United States). The ELISA kit for serotonin was obtained from LDN (BA E-5900R; Nordhorn, Germany). FITC-conjugated fibrinogen and Fura-2AM were from Molecular Probes (Eugene, OR, United States). Antibodies for protein detection: phospho-Akt, total-Akt, phospho-PI3K, total-PI3K, phospho-ERK, total-ERK, phospho-JNK, total-JNK, phospho-p38, total-p38, VASP ser^239^, VASP ser^157^, total-VASP, and β-Actin were acquired from Cell Signaling Technologies (Beverly, MA, United States). ERK inhibitor (PD98059), p38 inhibitor (SB203580), JNK inhibitor (SP600125), PI3K inhibitor (LY294002), and Rho kinase inhibitor (Y-27632) were acquired from Tocris Bioscience (Bristol, United Kingdom).

### 2.4 Animals

Seven-week-old male Sprague-Dawley rats and 6-week-old ICR mice were acquired from Orient Bio (Gyeonggi-do, Republic of Korea). The animals acclimated for 1 week in a pathogen-free facility (humidity of 50% ± 10%, temperature of 23°C ± 2°C) with a 12 h light-dark cycle. The experiments adhered to IACUC guidelines and received approval from the Ethics Committee of the College of Veterinary Medicine at Kyungpook National University (approval number: 2012-125). Animal procedures were conducted in compliance with humane practices.

### 2.5 Preparation of rat platelets

Blood was collected from rats via cardiac puncture using acid citrate dextrose solution as an anticoagulant. The collected blood was then centrifuged at 1,000 rpm for 7 min to obtain platelet-rich plasma (PRP). Next, the PRP was then centrifuged at 2,000 rpm for 7 min. The resulting pelleted platelets were resuspended in Tyrode’s buffer (137 mM NaCl, 12 mM NaHCO_3_, 5.5 mM glucose, 2 mM KCl, 1 mM MgCl_2_, and 0.3 mM NaHPO_4_, pH 7.4) to obtain washed platelets.

### 2.6 Platelet aggregation

Prepared washed platelets underwent platelet aggregation induced by agonists (collagen, thrombin or ADP). Platelet aggregation was detected using a light-transmission aggregometer from Chrono-log (Havertown, PA, United States). The platelets were treated with 1 mM of CaCl_2_, with or without GJ treatment, and pre-incubated for 1 min at 37°C before adding collagen (2.5 μg/mL), thrombin (0.1 U/mL) and ADP (10 μM). Aggregation was halted after 5 min. The experiments were repeated in triplicate with platelets from three different rats.

### 2.7 ATP and [Ca^2+^]_*i*_ mobilization assay

For ATP assay, platelet aggregation was conducted as described above. The platelets were collected and subjected to centrifugation at 7,500 rpm for 5 min. The supernatant was then collected for the ATP assay, following the manufacturer’s instructions. Intracellular calcium ion mobilization was detected using Fura-2AM. PRP was incubated with Fura-2AM. Next, platelets were pre-treated with 1 mM CaCl_2_, with or without GJ, and incubated for 1 min at 37°C before stimulation with collagen. Calcium mobilization was measured using a fluorescence spectrophotometer at 224 nm (F-2500; Hitachi, Tokyo, Japan). The experiments were repeated in triplicate from three different animals.

### 2.8 Fibrinogen binding of activated platelets

Platelets were incubated with GJ for 1 min before activation by collagen for an additional 5 min. We used FITC-conjugated fibrinogen to investigate the activation of integrin αIIbβ3. Afterward, the platelets were fixed with 0.5% paraformaldehyde and subjected to flow cytometry analysis using a BD FACS Aria^TM^III cell sorter (BD Biosciences, Franklin Lakes, NJ, United States). As a positive control, we treated platelets with 10 μM of EGTA. The experiments were repeated in triplicate from samples of three different animals.

### 2.9 Immunoblot analysis

Collagen-induced platelet aggregation was performed as mentioned above. Protein extraction from platelets utilized Pro-Prep (iNtRON Biotechnology, Gyeonggi-do, Republic of Korea). The protein concentration was determined using the Bradford method and normalized before loading onto a 10% SDS-PAGE. Subsequently, proteins were transferred to PVDF membranes and blocked with skim milk before incubation with the target primary antibodies (1: 3,000) at 4°C overnight. Membranes were incubated with secondary antibodies (1: 1,000) for 90 min at room temperature. Protein expressions were visualized using enhanced chemiluminescence in a developer (General Electrics, United States). The experiment was repeated in three biological replicates from three different rats.

### 2.10 ELISA assay

To investigate the secretion of TXB2 and serotonin, the assays were performed as described earlier in accordance to the manufacturer’s instructions (Irfan et al., 2021). Briefly, platelets suspension was immediately placed on ice after platelet aggregation assay. The supernatant was collected after centrifugation at 2,000 × *g* for 10 min at 4°C and analysed using ELISA assay. Intracellular cAMP and cGMP level detection was conducted as previously reported (Irfan et al., 2022). After platelet aggregation, the platelet mixture was immediately incubated on ice and then froze in −80°C. The next day, the mixture was thawed and centrifuged at 2,000 × *g* for 10 min at 4°C. The supernatant was obtained for ELISA assays, and were conducted following the manufacturer’s instructions. The plates were read at 420 nm on a microplate reader (Versamax; Molecular Devices, San Jose, CA, United States), as suggested in the manufacturer’s instructions. Experiments were repeated in triplicates from platelets of three different animals.

### 2.11 Clot retraction

Blood was collected from rats as described above and conducted as previously described (Irfan et al., 2022). Briefly, blood was centrifuged, and platelet-rich plasma (PRP) was separated. Extract of GJ and 10 μM of Y-27632 were added to the PRP before introducing 5 μL of RBCs. Thrombin was then added to make a final concentration of 1 U/mL and thoroughly mixed. The platelet mixture was then incubated for 2 h, and the thrombus weight was measured. The experiment was repeated using samples from three different animals.

### 2.12 Scanning electron microscopy

Following the platelet aggregation procedure described earlier, the platelets were directly fixed with 0.5% paraformaldehyde. The platelets were then oxidized using osmium tetraoxide and lyophilized before visualization using a field emission scanning electron microscope (SU8220; Hitachi, Tokyo, Japan). For platelet-neutrophil aggregate assays, cells were pre-treated with or without GJ for 1 min and activated with 2 μM of ADP. After 5-min incubation at room temperature, the cells were fixed, processed, and visualized as previously mentioned (Irfan et al., 2021).

### 2.13 Transmission electron microscopy

Platelets were treated with or without GJ, as mentioned above. Collagen was added to induce platelet aggregation for 30 s, followed by direct fixation using a solution of 2% glutaraldehyde and 2% paraformaldehyde in 0.2 M cacodylate buffer for 1 h at room temperature. The platelets were centrifuged at 5,000 rpm for 5 min and then washed with 0.1 M sodium cacodylate buffer. Secondary fixation involved 1% osmium tetraoxide in 0.1 M sodium cacodylate buffer, conducted at room temperature and shielded from light for 2 h. The fixed pellet was then washed with ultra-pure water and incubated overnight at 4°C with 3% UA-Zero EM Stain (a substitute for uranyl acetate from Agar Scientific, Stansted, ESS, United Kingdom) in 30% acetone. The fixed platelets were then dehydrated using increasing concentrations of acetone and epoxy (Sigma-Aldrich, St. Louis, MO, United States). Finally, the platelets were mixed with the epoxy mixture and dried in an oven for polymerization. The epoxy was then subjected to sectioning (EM UC7/FC7; Leica, Wetzlar, Germany) onto copper grids and visualized with a transmission electron microscope (HT 7700; Hitachi, Tokyo, Japan).

### 2.14 Flow cytometry analysis of platelet-neutrophil aggregates *ex vivo*

Whole blood was collected from 6-week-old ICR mice in Gyeonggi-do, Republic of Korea. These mice had acclimatized for a week in a pathogen-free facility with a 12-h light-dark cycle. Blood was harvested via cardiac puncture and stored in sodium citrate vacutainers (BD, Franklin Lakes, NJ, United States). Collected blood samples were pre-treated with or without GJ for 1 min before stimulation with 2 μM of ADP and were allowed to incubate for 5 min. The blood was then fixed with 1% paraformaldehyde before RBC lysis using ACK lysis buffer. Blood cells were stained with Alexa Fluor 488 conjugated CD41 (BioLegend, San Diego, CA, United States) and PE-conjugated Ly6G (BD, Franklin Lakes, NJ, United States). After staining, subpopulations of platelet-neutrophil aggregates were analyzed via flow cytometry (FACSAria^TM^III; BD Biosciences, Franklin Lakes, NJ, United States). Post-analysis was conducted using Flowlogic version 7 (Miltenyi Biotec, Bergisch Gladbach, Germany). The experiment was repeated in triplicates, from samples of three different mice.

### 2.15 Statistical analysis

We investigated the statistical significance of this study using GraphPad Prism 7.00 (San Diego, CA, United States). A one-way ANOVA, followed by a single-step Dunnett’s *post hoc* test was conducted. Normality of the dataset was tested using the D’Agostino and Pearson test. The data were presented as mean ± SD, and *P < 0.05* was considered significant.

## 3 Results

### 3.1 Metabolites of GJ extract identified by UPLC-QTOF-MS

**FIGURE 1:**
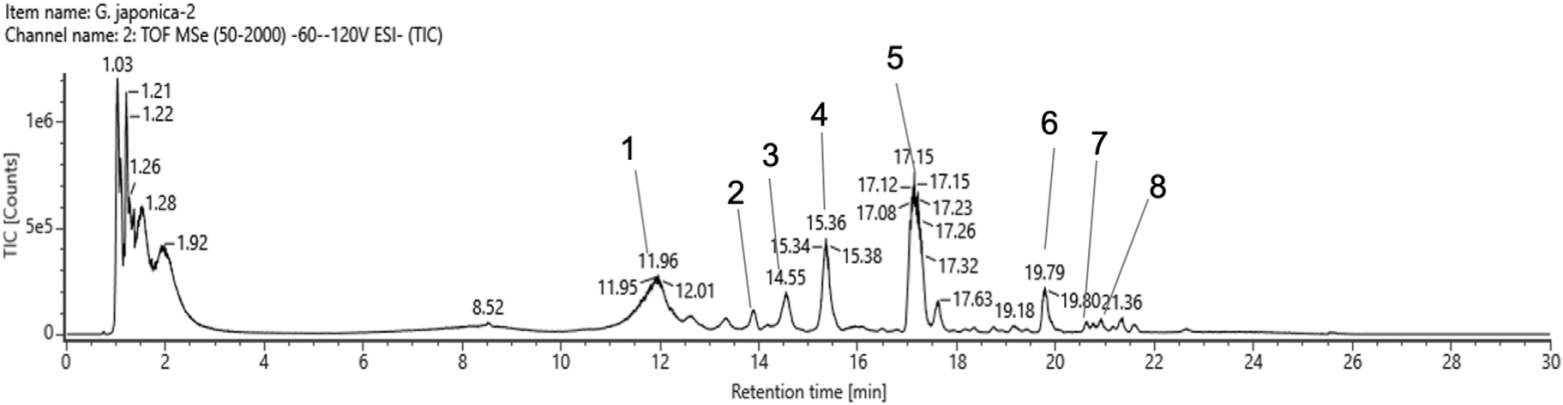
Total ion chromatogram spectrum of Geum Japonicum. Details of peaks identified and annotated are listed in Table 1.

Our analysis identified eight peaks, which were identified based on reference data from Yang et al. (2025) (Figure 1). Peaks 1, 3 and 4 were identified as 23-Hydroxytormentic acid (retention time; RT: 11.94, 14.56, 15.36 min). This compound is determined to be the major metabolite of GJ. The second most abundant metabolite is tormentic acid (RT: 17.12 min), followed by geumonoid (RT: 19.79 min). Other major metabolites include (10E,12Z)-9-Hydroxy-10,12-octadecadienoic acid (RT: 20.63 min) and pomolic acid (RT: 20.92 min). Peak 2, corresponding to 2α,3β,19α-trihydroxyurs-12-en-23,28-dioic acid, exhibited a relatively low detector count and was the least abundant among the six principal compounds (Table 1).

**TABLE 1:** Mass peaks identified by UPLC-QTOF-MS analysis of Geum Japonicum.

Peak	Component name	Neutral mass (Da)	Observed neutral mass (Da)	Observed m/z	Mass error (mDa)	Mass error (ppm)	Observed RT (min)	Detector count	Response	Adducts	Formula
1	23-Hydroxytormentic acid	504.34509	504.3451	503.3379	0.0	0.1	11.94	1,350,666	966,309	-H	C30H48O6
2	2α,3β,19α-Trihydroxyurs-12-en-23,28-dioic acid	518.32435	518.3227	517.3154	−1.7	−3.2	13.88	1,418	1,332	-H	C30H46O7
3	23-Hydroxytormentic acid	504.34509	504.3444	503.3371	−0.7	−1.4	14.56	402,381	283,704	-H	C30H48O6
4	23-Hydroxytormentic acid	504.34509	504.3449	503.3376	−0.2	−0.4	15.36	552,373	390,790	-H	C30H48O6
5	Tormentic acid	488.35017	488.3507	487.3435	0.6	1.1	17.12	1,969,842	1,398,793	-H	C30H48O5
6	Geumonoid	486.33452	486.3348	485.3275	0.3	0.6	19.79	408,851	289,185	-H	C30H46O5
7	(10E,12Z)-9-Hydroxy-10,12-octadecadienoic acid	196.23514	296.2336	295.2263	−1.5	−5.2	20.63	88,043	72,122	-H	C18H32O3
8	Pomolic acid	472.35526	482.3545	471.3472	−0.8	−1.6	20.92	99,603	71,264	-H	C30H48O4

### 3.2 GJ inhibits platelet aggregation induced by various agonists

**FIGURE 2:**
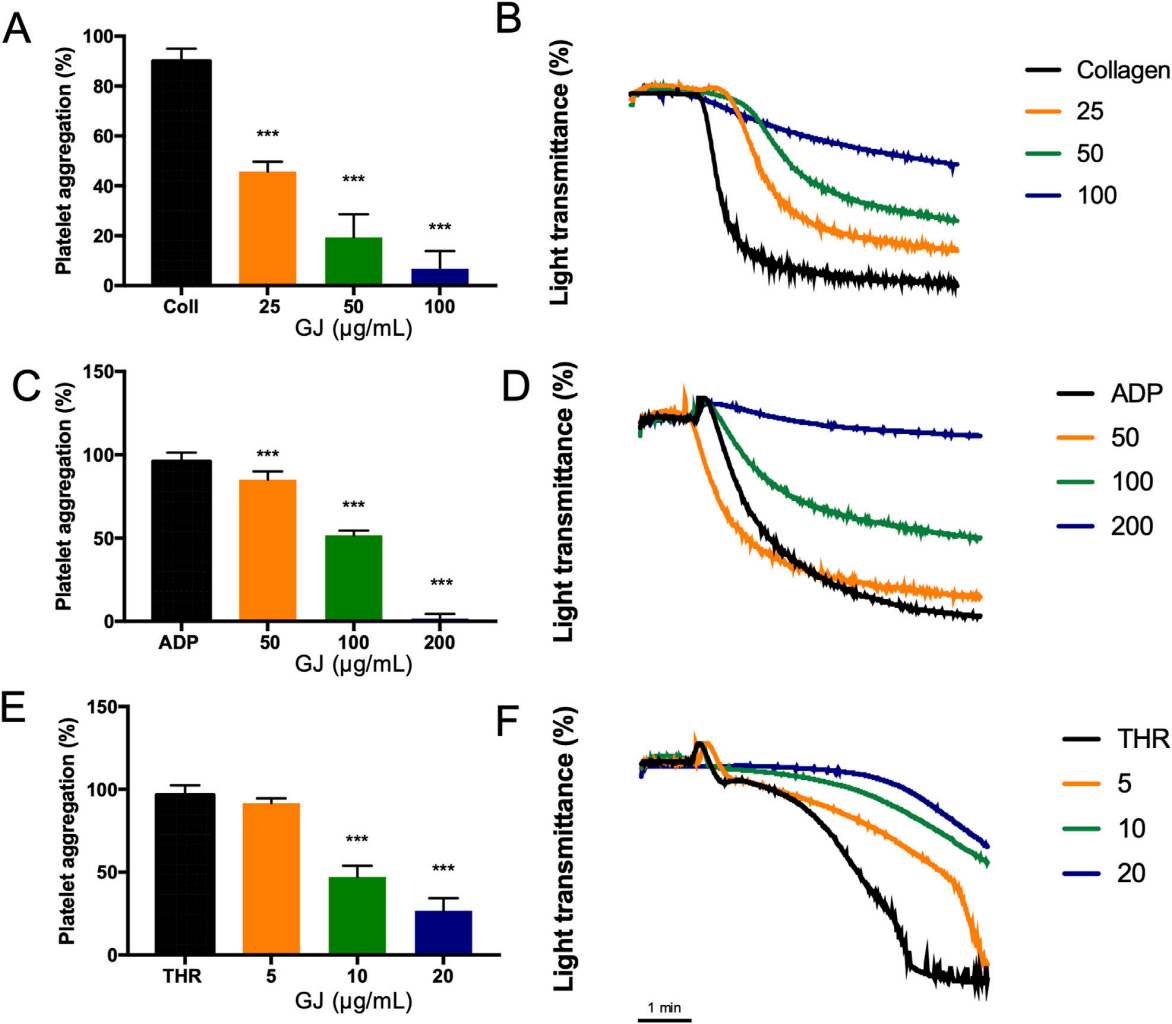
*Geum japonicum* (GJ) extract inhibits collagen, ADP, and thrombin-induced platelet aggregation. Platelet aggregation was induced by **(A)** collagen (2.5 μg/mL), **(C)** ADP (10 μM), and **(E)** thrombin (0.1 U/mL). Representative data of inhibition of platelet aggregation by GJ induced by collagen, ADP, and thrombin were shown in **(B, D, F)**, where the x-axis is time (minutes). Data were presented as mean ± SD. Experiments were repeated in triplicate, and ***P < 0.001 was considered statistically significant.

Various doses of GJ treatment inhibited aggregation induced by collagen (Figures 2A, B), ADP (Figures 2C, D), and thrombin (Figures 2E, F) in a dose-dependent manner. Notably, GJ most effectively inhibited thrombin-induced platelet aggregation, followed by collagen and ADP.

### 3.3 GJ inhibits granule secretion and reduces markers of platelet activation

**FIGURE 3:**
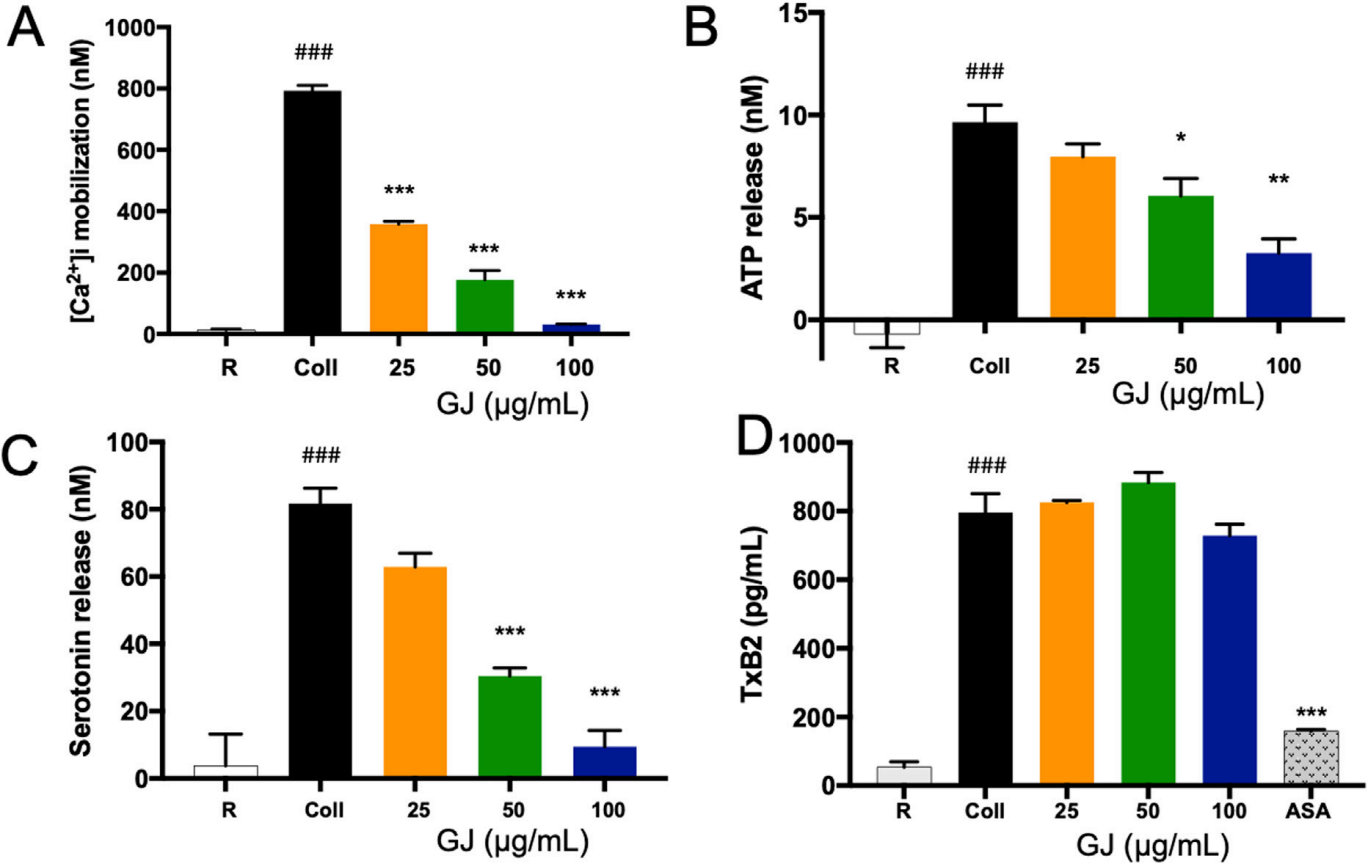
*Geum japonicum* extract prevented granule secretion in collagen-induced platelet aggregation. Fura-2AM was used to detect the mobilization of calcium ions in platelets when activated by collagen **(A)**. Platelet aggregation was induced by collagen, and the supernatant was collected to detect the secretion of ATP **(B)**, serotonin **(C)**, and TXB2 **(D)** according to the manufacturer’s instructions. Data were presented as mean ± SD. ASA, acetylsalicyclic acid. Experiments were repeated in triplicate, and *P < 0.05, **P < 0.01 and ***P < 0.001 were considered statistically significant against collagen-treated group. ^###^ *P* < 0.001 was considered significant comparing the collagen-treated group against the resting group.

Calcium ion mobilization is a marker of platelet activation, which has been detected to increase with stimulation by collagen. However, increasing doses of GJ effectively suppresses this response (Figure 3A). The activation of platelets causes the release of ATP, which has been increased with collagen. A 100 μg/mL dose of GJ significantly reduces ATP secretion (Figure 3B). Additionally, GJ suppresses serotonin secretion in stimulated platelets (Figure 3C). However, GJ does not impact the secretion of TXB2 (Figure 3D). Acetylsalicyclic acid (ASA) at 2.5 mg/mL was used as a positive control.

### 3.4 GJ increases the concentration cGMP and prevents platelet aggregation via inhibition of integrin αIIbβ3

**FIGURE 4:**
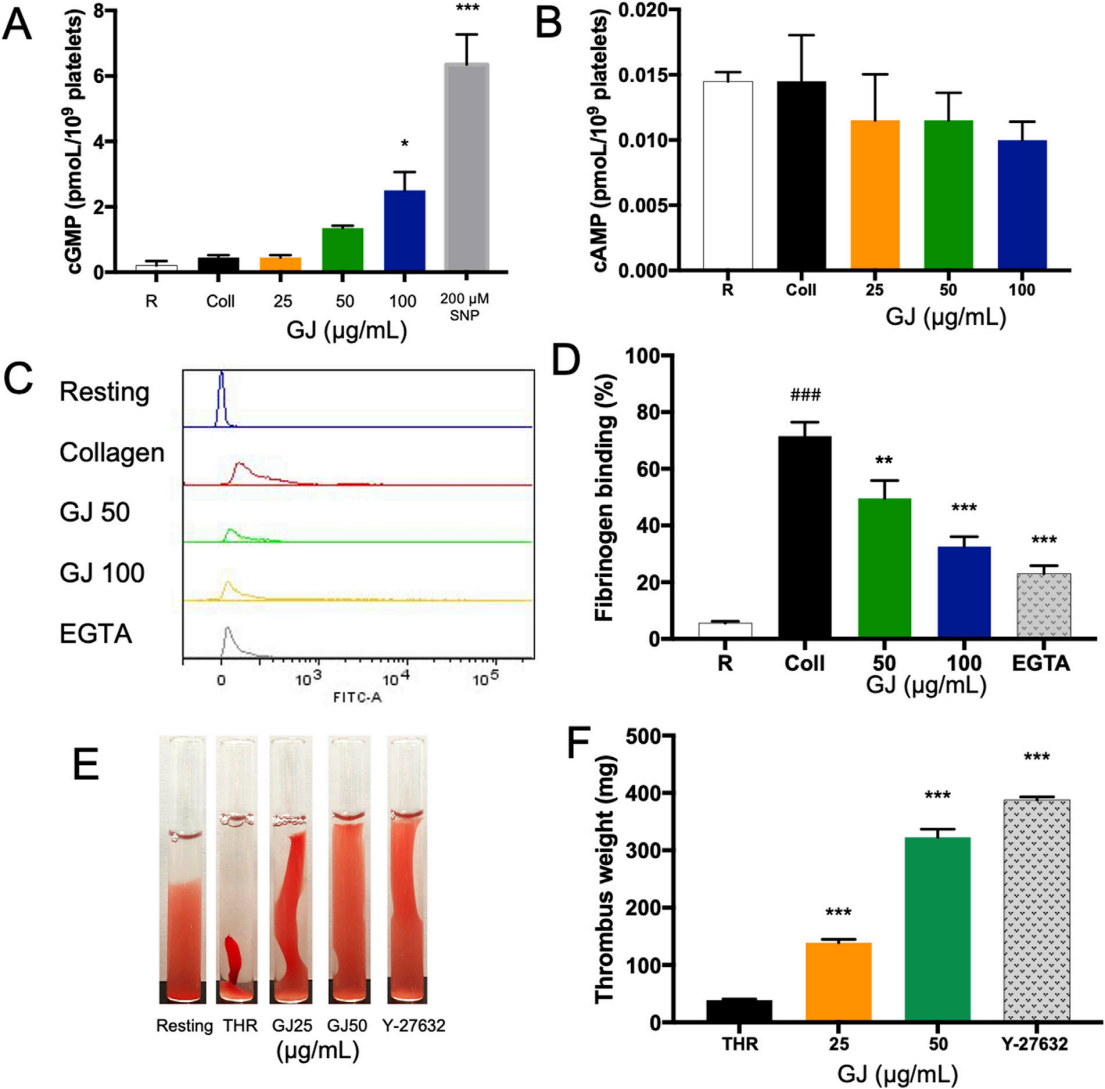
*Geum japonicum* (GJ) enhanced the concentration of cGMP to inhibit platelet aggregation. Platelet aggregation was induced by collagen, and the supernatant was collected. The concentration of cGMP **(A)** and cAMP **(B)** were detected via ELISA assay. Platelets were treated with GJ and 10 μM of EGTA as a positive control, activated with collagen and FITC-conjugated fibrinogen to detect platelet activation via integrin αIIbβ3 via flow cytometry **(C)**. The experiment was repeated three times, and the average was taken **(D)**. The ability of GJ to inhibit the outside-in signaling of αIIbβ3 was investigated via clot retraction assay **(E)**, and the weight of the thrombus was weighed after 2 h of incubation **(F)**. A dose of 10 μM of Y-27632 was used as a positive control. Data were presented as mean ± SD, and **P < 0.01 and ***P < 0.001 were considered statistically significant. Experiments were conducted in triplicate. ^###^ *P* < 0.001 was considered significant comparing the collagen-treated group against the resting group.

We investigated the intracellular concentration of cAMP and cGMP, essential in regulating platelet aggregation. Our findings showed that 100 μg/mL of GJ has significantly increased cGMP concentration (Figure 4A), while the concentration of cAMP remains unchanged (Figure 4B). This indicates that GJ exhibits its anti-platelet activity via the upregulation of cGMP rather than cAMP. The conformational change of integrin αIIbβ3 is vital for platelet aggregation and activation. Fibrinogen binds to αIIbβ3 on platelets, allowing them to form aggregates. Utilizing FITC-conjugated fibrinogen, we detected αIIbβ3 activation via inside-out signaling. We found that the percentage of fibrinogen that was bound increased in collagen-stimulated platelets, and this was decreased with 50 and 100 μg/mL of GJ. EGTA (10 μM) was a positive control (Figures 4C, D). The outside-in activation of αIIbβ3 activates the Rho kinase pathway, which is indicated by clot retraction. GJ significantly inhibited thrombin-induced clot retraction at 25 and 50 μg/mL (Figure 4E). The amount of trapped plasma indicates the rigidity of the fibrin mesh and is indicated by the weight of the thrombus (Figure 4F).

### 3.5 GJ inhibits protein expressions of genes related to platelet activation

**FIGURE 5:**
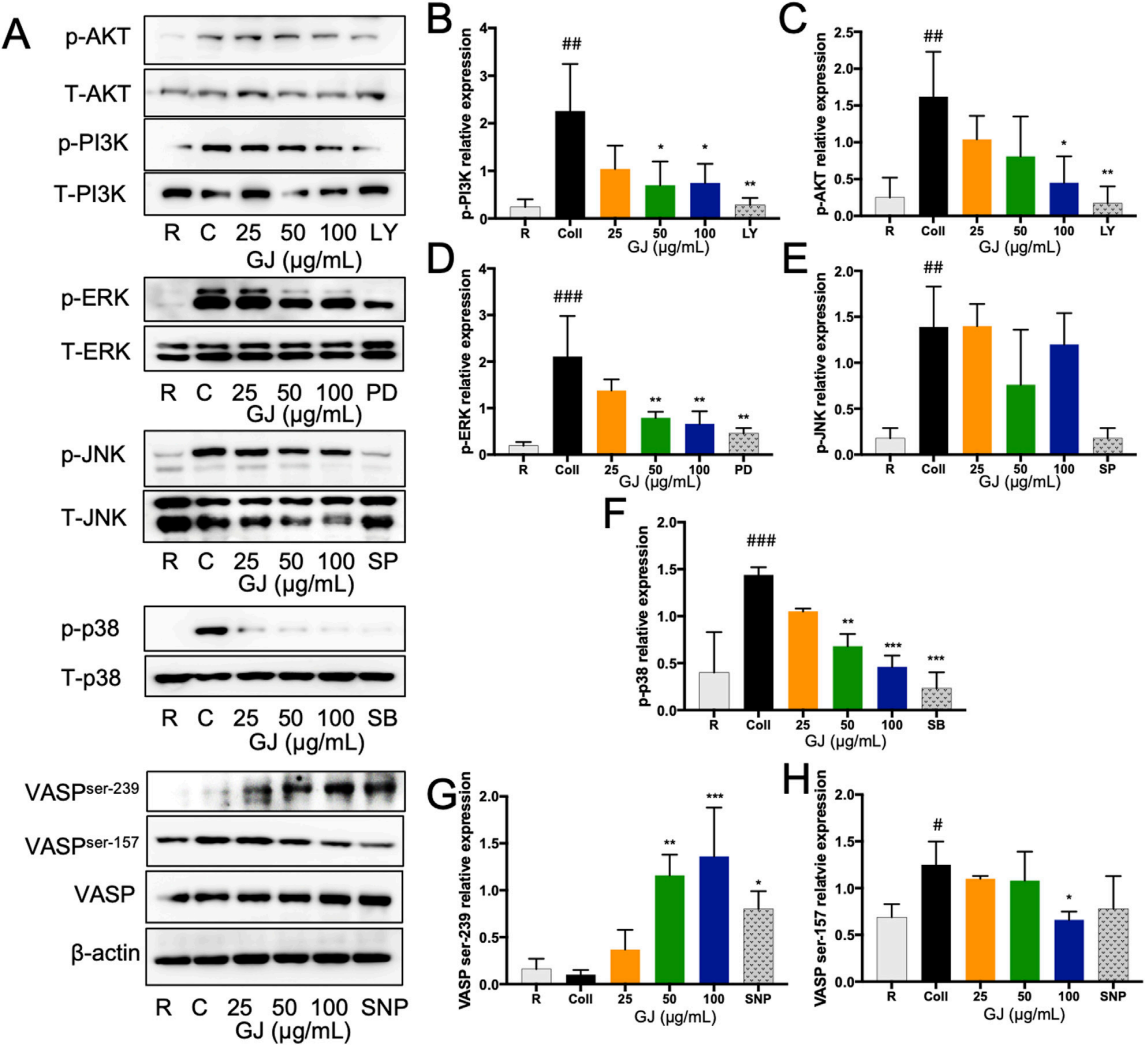
*Geum japonicum* inhibits platelet aggregation by inhibiting PI3K/Akt and MAPK pathways. Platelets were induced aggregation with collagen, and the platelets were collected. Protein was extracted, and the expression of proteins related to platelet activation was investigated. Representative blots were presented in **(A)**. The expression of phospho-PI3K **(B)**, phospho-Akt **(C)**, phospho-ERK **(D)**, phospho-JNK **(E)** and phospho-p38 **(F)** were normalized against their total forms. Expression of VASPser^239^ **(G)** and VASPser^157^ **(H)** were normalized against the total form of VASP. β-Actin was used as a housekeeping gene. The experiment was conducted in triplicate, and the blots were quantified using ImageJ. ERK inhibitor (PD98059), p38 inhibitor (SB203580), JNK inhibitor (SP600125), and PI3K inhibitor (LY294002) were used as a positive control. Data were presented as mean ± SD, and *P < 0.05, **P < 0.01 and ***P < 0.001 was considered statistically significant against collagen-treated group. ^#^ *P* < 0.05, ^##^ *P* < 0.01 and ^###^ *P* < 0.001 were considered statistically significant against the resting group. Experiments were conducted in triplicate. R, Resting, C, collagen.

The PI3K/Akt and MAPK pathways are associated with platelet activation. Representative blots are shown in Figure 5A. Our findings show that the expression of p-PI3K and p-Akt were significantly suppressed by GJ (Figures 5B, C). Protein expressions of p-ERK and p-p38 were also significantly inhibited by GJ but not p-JNK (Figures 5D–F). The protein expressions of VASP ser239 and VASP ser157 were investigated to explore the role of cGMP and cAMP further. Our findings show that collagen-treatment increased expression of VASP ser^157^ and was suppressed with GJ treatment. GJ treatment has also upregulated VASP ser^239^ expression (Figures 5G, H). The relative expressions of the blots were normalized against their total forms. *β-Actin* was used as a housekeeping gene. This confirms that GJ mainly prevents platelet aggregation via the cGMP pathway and inhibits proteins in the PI3K/Akt and MAPK pathways.

### 3.6 GJ prevents platelet shape change and secretion of granules

**FIGURE 6:**
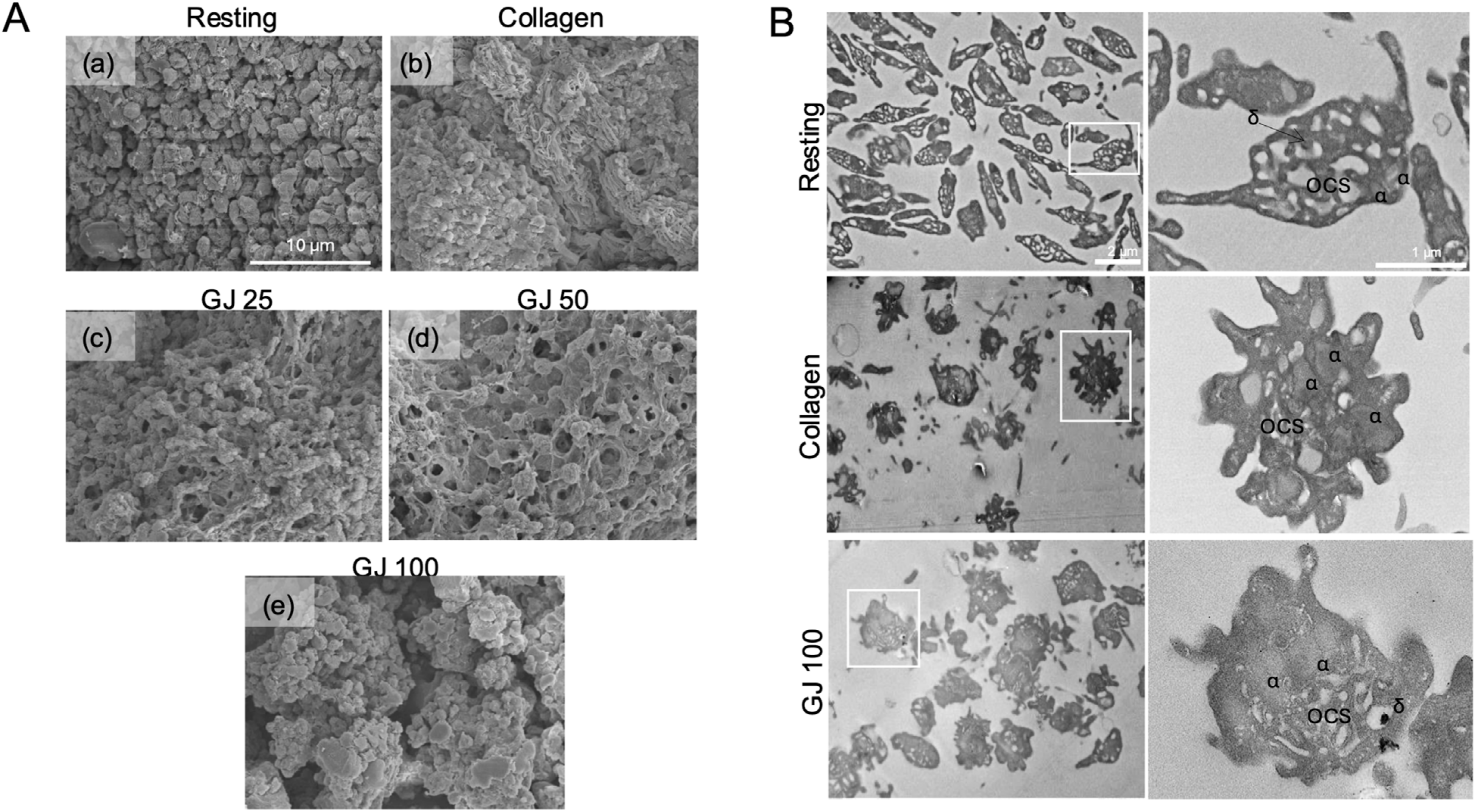
*Geum japonicum* (GJ) prevented platelet aggregation, shape change, and degranulation. **(A)** Platelet aggregation was conducted, fixed, and oxidized using osmium tetraoxide. Scanning electron microscopy was used to investigate the shape of platelets in resting state (a), collagen (b), 25 μg/mL of GJ (c), 50 μg/mL of GJ (d) and 100 μg/mL of GJ (e). GJ extract prevents granule secretion in collagen-induced platelet aggregation **(B)**. Platelets were treated with or without GJ, followed by activation with collagen for 30 s, directly fixed before embedding in epoxy, and sectioned for visualization using transmission electron microscopy. Sections of resting and activated platelets were observed at a magnification of ×2.5k (left panel) and ×10.0k (right panel). α, alpha granules; δ, dense granules; OCS, open canalicular system.

As platelet shape change is a vital step in the activation of platelets, scanning electron microscopy was used to observe the morphology of platelets (Figure 6A). With activation by collagen, the platelets appeared fibrous (Figure 6A[b]) as compared to platelets in the resting state (Figure 6A[a]). With treatment of increasing doses of GJ (25, 50 and 100 μg/mL), platelets appeared less activated as compared to when stimulated with collagen (Figure 6A[c]–[e]). Transmission electron microscopy was used to observe the intracellular structure of platelets (Figure 6B). From our findings, the activation of platelets with collagen caused degranulation of platelets, whereas treatment of GJ prevented this from happening. Alpha granules (α) and dense granules (δ) were identified. The open canalicular system (OCS) that transports granules from the platelets was visible in resting platelets but filled with granules in the collagen-stimulated group. The OCS was then observable after GJ treatment. This further confirms that the treatment of GJ has prevented the activation and degranulation of platelets.

### 3.7 GJ prevented platelet aggregation via the enhancement of cGMP

**FIGURE 7:**
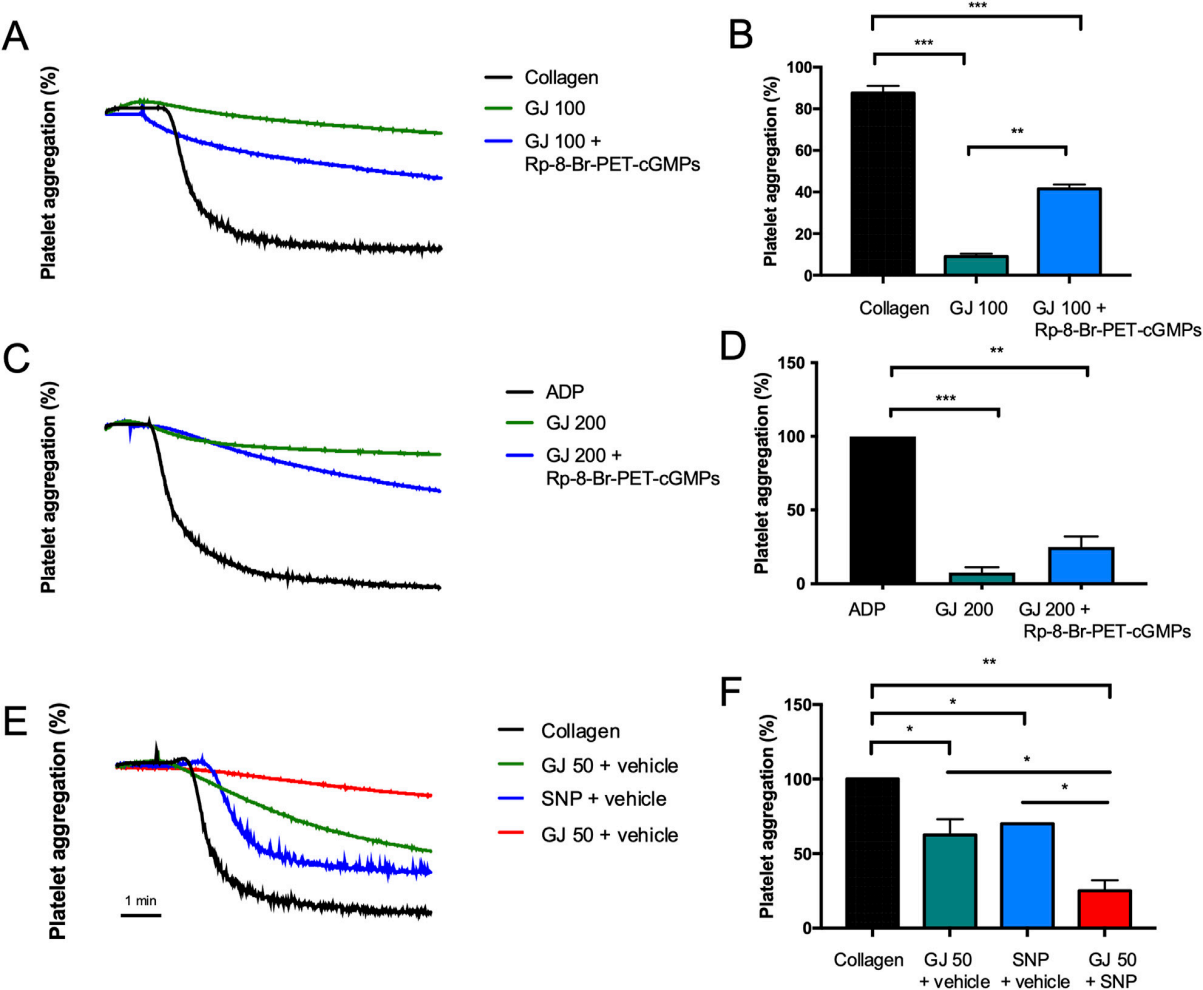
*Geum japonicum* (GJ) inhibits platelet aggregation via enhancement of cGMP expression. Platelets were treated with GJ and 25 μM of Rp-8-Br-PET-cGMPs (cGMP inhibitor). Platelets were then induced with collagen **(A, B)** and ADP **(C, D)**. Platelets were treated with GJ, with or without 200 μM of sodium nitroprusside (SNP; cGMP agonist) and stimulated with collagen **(E, F)**. The x-axis for platelet aggregation curves is in time (minutes). Experiments were repeated three times, and the data were presented as mean ± SD and *P < 0.05, **P < 0.01 and ***P < 0.001 were considered statistically significant.

A cGMP inhibitor (Rp-8-Br-PET-cGMPs) and agonist (200 μM of SNP) were used to elucidate the effects of GJ via the cGMP pathway. From our findings, 100 μg/mL of GJ has significantly inhibited platelet aggregation but treatment with the cGMP inhibitor (Rp-8-Br-PET-cGMPs) reduced the efficacy of GJ (Figures 7A, B). This indicates that GJ elevates cGMP to prevent platelet aggregation. In ADP-induced platelet aggregation, an inhibitor of cGMP slightly reduced GJ’s anti-platelet effect, which was not statistically significant (Figures 7C, D). Using an agonist of cGMP (SNP), a combination of GJ and SNP has demonstrated increased anti-platelet activity (Figures 7E, F). This further confirms that GJ acts via the elevation of cGMP to inhibit platelet aggregation. However, the inhibitor of cGMP did not wholly attenuate the ability of GJ to inhibit platelet aggregation. This indicates the anti-platelet mechanism of GJ involves upregulating cGMP and other pathways, such as the PI3K/Akt and MAPK pathways (Figure 5).

### 3.8 GJ inhibits the formation of platelet-neutrophil aggregates *ex vivo*

**FIGURE 8:**
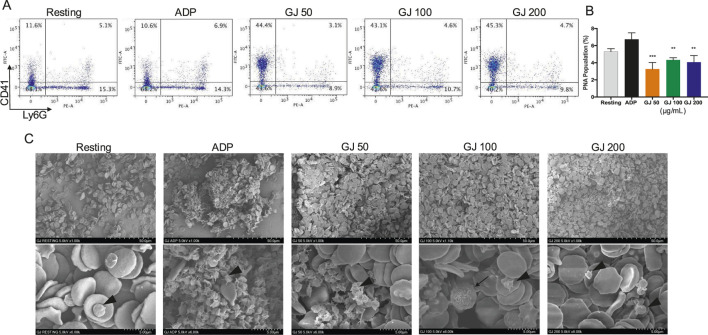
*Geum japonicum* (GJ) inhibits the formation of ADP-induced platelet-neutrophil aggregate formation. Whole blood was collected from 6-week-old ICR mice. Platelet aggregation was induced with ADP in the presence of blood cells with or without treatment of GJ. The cells were then fixed, stained, and analyzed with a flow cytometer. Representative quadrants of each treatment group were presented in **(A)**. Quantitative data (n = 3) were presented in **(B)**. Data were presented as mean ± SD, and *P < 0.05, **P < 0.01 and ***P < 0.001 were considered statistically significant. **(C)** Scanning electron microscopy was conducted after fixing cells treated with or without GJ for 1 min, followed by stimulation with ADP. Platelets were indicated with arrowheads, and leukocytes were indicated with arrows. The top row of images was visualized at ×1.00k, whereas the bottom row was imaged at ×6.00k.

As the formation of PNAs contributes to thromboinflammatory diseases, we investigate whether GJ can inhibit the formation of PNAs. Our results show that ADP significantly increased the population of PNAs in the whole blood of mice, where it is decreased by treatment of GJ (Figure 8A), where the quantitative data of three technical replicates were presented in Figure 8B. We observed an increase in platelet population in the whole blood of mice (top left quadrant, CD41) treated with GJ compared to the control and ADP-treated groups. With SEM, we observed ADP-induced platelet activation with early signs of platelet aggregation. We observed that with 100 and 200 μg/mL of GJ treatment, platelets (indicated with red arrowheads) are at the early stages of platelet aggregation, identified by the early formation of pseudopodia. In contrast, leukocytes (marked with a red arrow) were not attached to platelets (Figure 8C).

## 4 Discussion

Blood vessel injury causes the release of collagen which binds to the GPVI receptor of platelets, causes their activation (Poulter et al., 2017). The P2Y12 receptor, a known receptor for ADP, is a target of antithrombotic thienopyridines like clopidogrel and ticlopidine (Soulet et al., 2004). Thrombin can bind to the protease-activated receptors 1 and 4, and the GPIb-V-IX complex on platelets (De Candia, 2012). In this study, we utilized these agonists to investigate the efficacy of GJ. Our findings show that GJ effectively inhibits platelet aggregation induced by these agonists. In another review, collagen activates the GPIb and GPIIb/IIIa receptors on platelets at high blood flow shear with the help of vWF and GPIa/IIa and GPVI at low shear. This causes the release of ADP and thromboxane A2 (TXA2), inducing procoagulant activity by releasing calcium ions and further causing platelet aggregation and spreading. The release of ADP causes platelet shape change and aggregation (Jennings, 2009).

Following stimulation by collagen, phospholipase C is activated, which triggers calcium to move out from the dense tubular system. A high intracellular calcium ion level causes granule secretion and the activation of phospholipase A2 (PLA2). The PLA2 pathway involves the activation of arachidonic acid that will cause the secretion of TXA2 that will be directly converted into TXB2 (Sangkuhl et al., 2011). In our study, GJ inhibited the extracellular mobilization of calcium ions and the secretion of ATP and serotonin, released by dense granules. However, GJ did not inhibit the levels of TXB2, where ASA was used as a positive control (ASA targets COX-1 to inhibit TXA2 secretion) (Lucotti et al., 2019). Our findings reveal the specificity of GJ to inhibit the secretion of dense granules but do not target the TXA2 pathway.

Activation of the GPIb-IX-V and GPVI by collagen will activate the PI3K pathway and the activation of the PLCγ pathway that leads to the conformational change of the integrin αIIbβ3—regarded as the inside-out signaling, which plays a vital role in the activation of platelets (Huang et al., 2019). The outside-in signaling of integrin αIIbβ3 can be activated by fibrinogen, and it activates the Src-family kinases (SFKs), PLCγ2, focal adhesion kinase (FAK), and RhoA. RhoA is essential to platelet shape change and clot retraction. This further activates the Rho-associated kinase and myosin light chain phosphorylation (Durrant et al., 2017). In our study, GJ has shown effective inhibition of the integrin αIIbβ3 via the inside-out and outside-in signaling, playing a role in preventing platelet aggregation and thrombus formation. As RhoA is vital for platelet shape change, we have investigated the shape of platelets via SEM and TEM. Treatment of GJ has evidently prevented platelet shape change induced by collagen (Figure 6A) and prevented the degranulation of platelets, as observed by TEM (Figure 6B). Moreover, our results show that GJ reduced the population of ADP-induced PNAs in whole blood of mice (Figure 8A). We hypothesize that GJ reduced secretion of P-selectin stored in α-granules of platelets. This reduces the availability of P-selectin to bind with P-selectin glycoprotein ligand 1 (PSGL-1) on neutrophils to form PNAs (Mauler et al., 2016). This should be validated in future studies and its mechanism of action should be studied in detail.

Yang et al. (2025) characterized and identified metabolites of GJ in rats that were given gavage of GJ using ultra-high-performance liquid chromatography coupled with quadropole Exactive Orbitrap mass spectrometry (Yang et al., 2025). In our UPLC-QTOF-MS analysis, 86 of the 94 metabolites that were identified by Yang et al. (2025) was analyzed. We identified 23-hydroxytormentic acid as the most abundant metabolite, followed by tormentic acid (Table 1). Yang et. al’s (2025) identified seven GJ-derived metabolites in the plasma and heart tissue of rats, suggesting their potential involvement in the treatment of cardiovascular diseases. Among these, 23-hydroxytormentic acid is one of the key metabolites. Yang et al’s (2025) findings suggest that 23-hydroxytormentic is bioavailable *in vivo* and our study suggests that 23-hydroxytomentic acid exhibit anti-platelet activity *ex vivo*. Collectively, GJ exhibits promising potential as an antiplatelet agent with possible applications in treatment of cardiovascular disease.

GJ has been previously reported for its therapeutic effects in the reconstitution of coronary vasculature (Chen et al., 2014). GJ also exhibit antioxidant activity (Lee et al., 2007), which may potentially assist in preventing the progression of atherosclerosis as ROS causes damage in the vascular endothelium. Moreover, 3,4,5-trihydroxybenzaldehyde (also known as gallic aldehyde) from GJ has inhibited MMP-9 in human aortic smooth muscle cells, which plays a vital role in the progression of atherosclerotic lesions (Suh et al., 2008). Tannins isolated from GJ were also previously reported to exhibit anticoagulant properties by prolonging clotting time in rabbit plasma (Dong et al., 1998). Metabolites of ethyl gallate include gallic aldehyde. The metabolite 1,2,3-benezetriol that was identified in our GCMS analysis, also known as pyrogallol, is a possible product of gallic aldehyde decarboxylation (Supplementary Figure 1). Pyrogallol was reported for its anti-inflammatory effects in herbal extracts (Nicolis et al., 2008; Chantarasakha et al., 2022). In recent years, platelet activation has been common in thromboinflammatory diseases, especially because of its role in recruiting leukocytes to the site of inflammation (Jenne and Kubes, 2015). Pyrogallol is a polyphenol that generates the superoxide anion. Although it is a radical-generating polyphenol, and while a previous study has shown that pyrogallol induces apoptosis in human platelets (Bruges et al., 2018), the accumulation of superoxide anion depletes superoxide dismutase (SOD) to be converted into hydrogen peroxide and water by glutathione peroxidase and catalases (Wang et al., 2018). It was also shown that SOD induces platelet activation in collagen and arachidonic acid primed-human platelets (Iuliano et al., 1991), proposing that pyrogallol-limited SOD will potentially prevent platelet activation. Our study suggests pyrogallol is a potential GJ metabolite exhibiting anti-platelet activity. Pan-assay interference compounds (PAINS) are shown to generate false positives in biological assays by interfering with assay components. Common PAINS include rhodanines, quinones, enones and catechols (Bael et al., 2014). Pyrogallol, detected in our GCMS analysis, has a catechol-like moiety reported to interfere with certain biological assays due to its redox activity (Marklund and Marklund, 1974). This property warrant caution when interpreting its biological relevance in translational research.

## 5 Conclusion

In a nutshell, our study has shown the mechanism of action of GJ in rat platelets; GJ inhibited collagen, ADP and thrombin-induced platelet aggregation, inhibited granule secretion (but not secretion of TXB2), inhibition of the proteins in the PI3K/Akt and MAPK pathway, prevented platelet shape change and acts via the regulation of cGMP to prevent platelet aggregation and prevented formation of PNAs. Further studies should be conducted *in vivo* to further elucidate the pathway of cGMP activation involving PKG, and its downstream pathways. The effect of GJ should be further validated in the thrombin-induced platelet aggregation pathway. Human platelets should be utilized to study GJ-mediated cGMP activation that exhibits anti-platelet activity and future studies on the pharmacokinetics of GJ is needed to elucidate the physiological relevance of doses of GJ used in this study. The underlying mechanism of how GJ exacerbates PNA formation should also be elucidated. Our study further suggests that 23-hydroxytormentic acid possesses antiplatelet activity. A key limitation of our study is the reliance on *ex vivo* experiments conducted in primary rat platelets. Our findings may not fully reflect the pharmacological effects following oral administration of GJ, due to metabolic modifications *in vivo*. However, our study suggests that GJ is a suitable candidate for development as a cardiovascular disease supplement as it has been reported to have therapeutic effects against various aspects of cardiovascular disease.

## Data Availability

The original contributions presented in the study are included in the article/Supplementary Material, further inquiries can be directed to the corresponding author.
