# Supplementary material for: Geum japonicum Thunb. exhibits anti-platelet activity via the regulation of cyclic guanosine monophosphate
Source: Front Pharmacol. 2025 Jun 26;16:1538417. doi: 10.3389/fphar.2025.1538417 (PMC12240943; doi:10.3389/fphar.2025.1538417)
Supplement: Supplementary file 1 [file DataSheet1.docx]

**Supplementary Material**

**Methods**

**Gas chromatography-mass spectrometry (GC-MS) analysis of GJ**

The GC-MS analysis was performed using an Agilent 7890A GC instrument (Agilent Technologies, Santa Clara, CA, USA). The instrument featured a 30 m × 0.25 mm (DB-5MS) chromatography column and an Agilent 5975C mass selective detector. The extract was injected at a temperature of 250°C. Source and transfer line temperatures were set at 230°C and 280°C, respectively. Initially, the column temperature was 70°C for 1 min, followed by a gradual increase at 5°C/min to a final temperature of 300°C, maintained for 30 min. Mass spectrometry data were obtained in scan and electron ionization modes to analyze the metabolites of GJ.


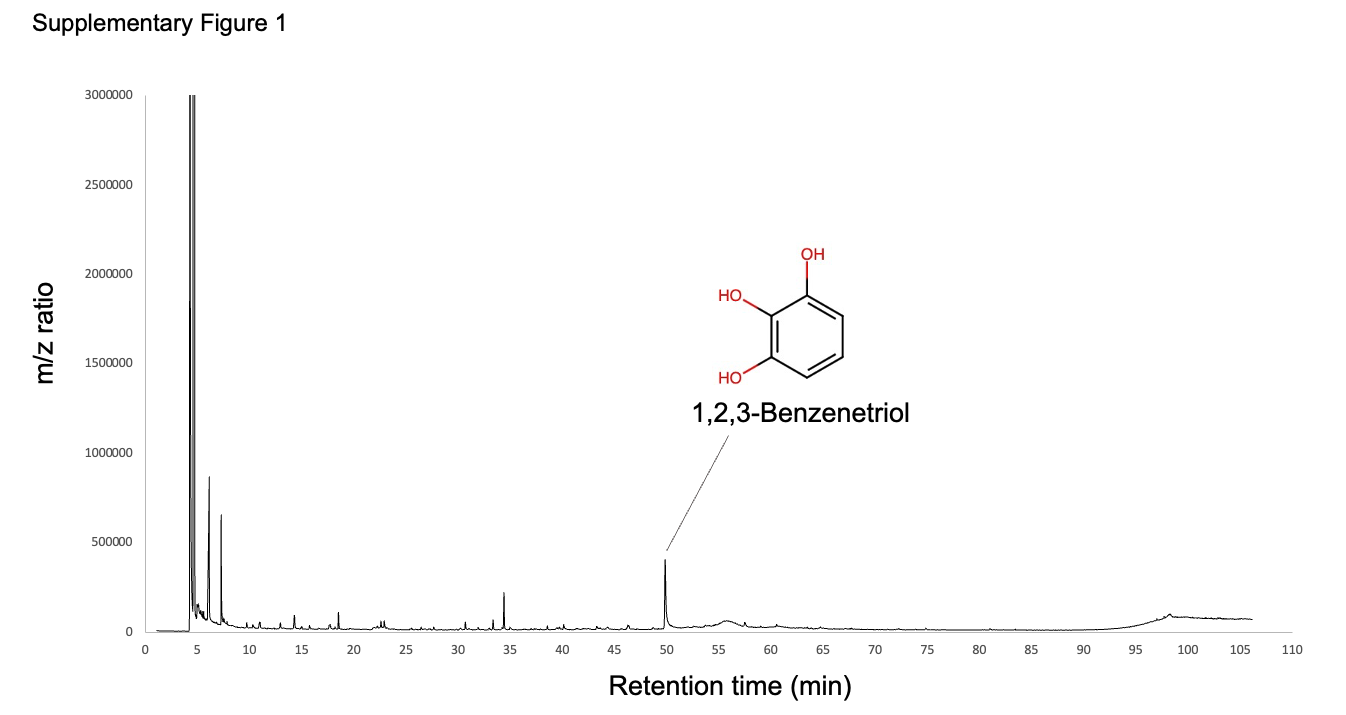


**Supplementary Figure 1.** Abundant metabolites in *Geum japonicum* extract were identified using GCMS analysis.

**Results**

Metabolites of GJ detected from GCMS analysis were presented in Figure 1. The major abundant compound in GJ detected is 1,2,3-Benezetriol (C6H6O3; RT: 49.87 min; CAS no.: 87-66-1) as shown in Figure 1. Other peaks in the chromatogram were compounds degraded from the extraction solvent.
